# An *In-Silico*, *In-Vitro* and *In-Vivo* Combined Approach to Identify NMNATs as Potential Protein Targets of ProEGCG for Treatment of Endometriosis

**DOI:** 10.3389/fphar.2021.714790

**Published:** 2021-10-14

**Authors:** Sze Wan Hung, Bo Liang, Yating Gao, Ruizhe Zhang, Zhouyurong Tan, Tao Zhang, Pui Wah Jacqueline Chung, Tak Hang Chan, Chi Chiu Wang

**Affiliations:** ^1^ Department of Obstetrics and Gynaecology, The Chinese University of Hong Kong, Hong Kong, China; ^2^ Guangdong Provincial Key Laboratory of Marine Biotechnology, Institute of Marine Sciences, Shantou University, Shantou, China; ^3^ Center for Reproductive Medicine, Henan Key Laboratory of Reproduction and Genetics, The First Affiliated Hospital of Zhengzhou University, Zhengzhou, China; ^4^ Department of Applied Biology and Chemical Technology, State Key Laboratory of Chemical Biology and Drug Discovery, The Hong Kong Polytechnic University, Hong Kong, China; ^5^ Department of Chemistry, McGill University, Montreal, QC, Canada; ^6^ Reproduction and Development, Li Ka Shing Institute of Health Sciences, The Chinese University of Hong Kong, Hong Kong, China; ^7^ School of Biomedical Sciences, The Chinese University of Hong Kong, Hong Kong, China; ^8^ Chinese University of Hong Kong-Sichuan University Joint Laboratory in Reproductive Medicine, The Chinese University of Hong Kong, Hong Kong, China

**Keywords:** antioxidants, endometriosis, treatment, prodrug, EGCG, protein targets, NMNATs, reactive oxygen species

## Abstract

Endometriosis is defined as endometrial tissues found outside the uterine cavity. ProEGCG is a prodrug of Epigallocatechin gallate (EGCG), a potent polyphenol found in green tea. It inhibits the development of endometriotic lesions of mouse model *in vivo*, with higher efficacy and more remarkable anti-oxidative ability than EGCG. Our study aims to identify the molecular binding targets and pharmacological actions of ProEGCG in treating endometriosis. Protein target interaction study is essential to fully characterize the mechanism of actions, related therapeutic effects, and side effects. We employed a combined approach, starting with an *in silico* reverse screening of protein targets and molecular docking, followed by *in vitro* cellular thermal shift assay (CESTA) to assess the stability of protein-small molecule complexes. Then microarray and immunostaining of endometriotic lesions in mice *in vivo* confirmed the molecular interaction of the selected targets after treatment. Our study identified enzymes nicotinamide nucleotide adenylyltransferase (NMNAT)1 and NMNAT3 as protein targets of ProEGCG *in silico* and *in vitro* and were overexpressed after ProEGCG treatment *in vivo*. These findings suggested that participation in nicotinate and nicotinamide metabolism potentially regulated the redox status of endometriosis *via* its antioxidative capacities through binding to the potential therapeutic targets of ProEGCG.

## 1 Introduction

Endometriosis is an estrogen-dependent gynecological disorder. It is a benign condition defined as endometrial tissues found outside the uterine cavity, usually in ovaries or fallopian tubes (Burney and Giudice, 2012; Zondervan et al., 2020). The occurrence is around 5–10% in reproductive women (Zondervan et al., 2018; Zondervan et al., 2020), while predominant clinical presentations are pain and infertility. Etiology of endometriosis includes Sampson’s theory of retrograde menstruation, which describes the dissemination of endometrium into the peritoneal cavity through fallopian tubes during menstruation (Sampson, 1927). Endometriosis is a progressive disease of oxidative stress and chronic inflammation (Gordts et al., 2017).

Nowadays, the choice of current treatment for endometriosis depends on patients’ medical history, fertility plan and severity of symptoms. Widely used medications include gonadotrophin-releasing hormone analogues (GnRH) and combined oral contraceptives. The effectiveness of different treatments varies, and it also comes with side effects. (Zondervan et al., 2020; Hung et al., 2021). Conservative or definitive surgery either has a high recurrence rate or may cause sterility (Valle and Sciarra, 2003; Falcone and Flyckt, 2018). Therefore, ideal new treatment and novel drug discovery are needed to help women alleviate the symptoms and cure the disease. In preclinical research of a new drug, it is imperative to understand the mechanism of the drugs’ actions.

During the early stage of endometriosis, macrophages and retrograded endometrial tissues in the peritoneal cavity increase reactive oxygen species (ROS) level, and induce oxidative stress and inflammatory reaction, which favor the growth of the endometriotic cells (Zeller et al., 1987). An imbalance between ROS and antioxidants contributes to the progression of endometriotic lesions (Szczepanska et al., 2003).

**FIGURE 1:**
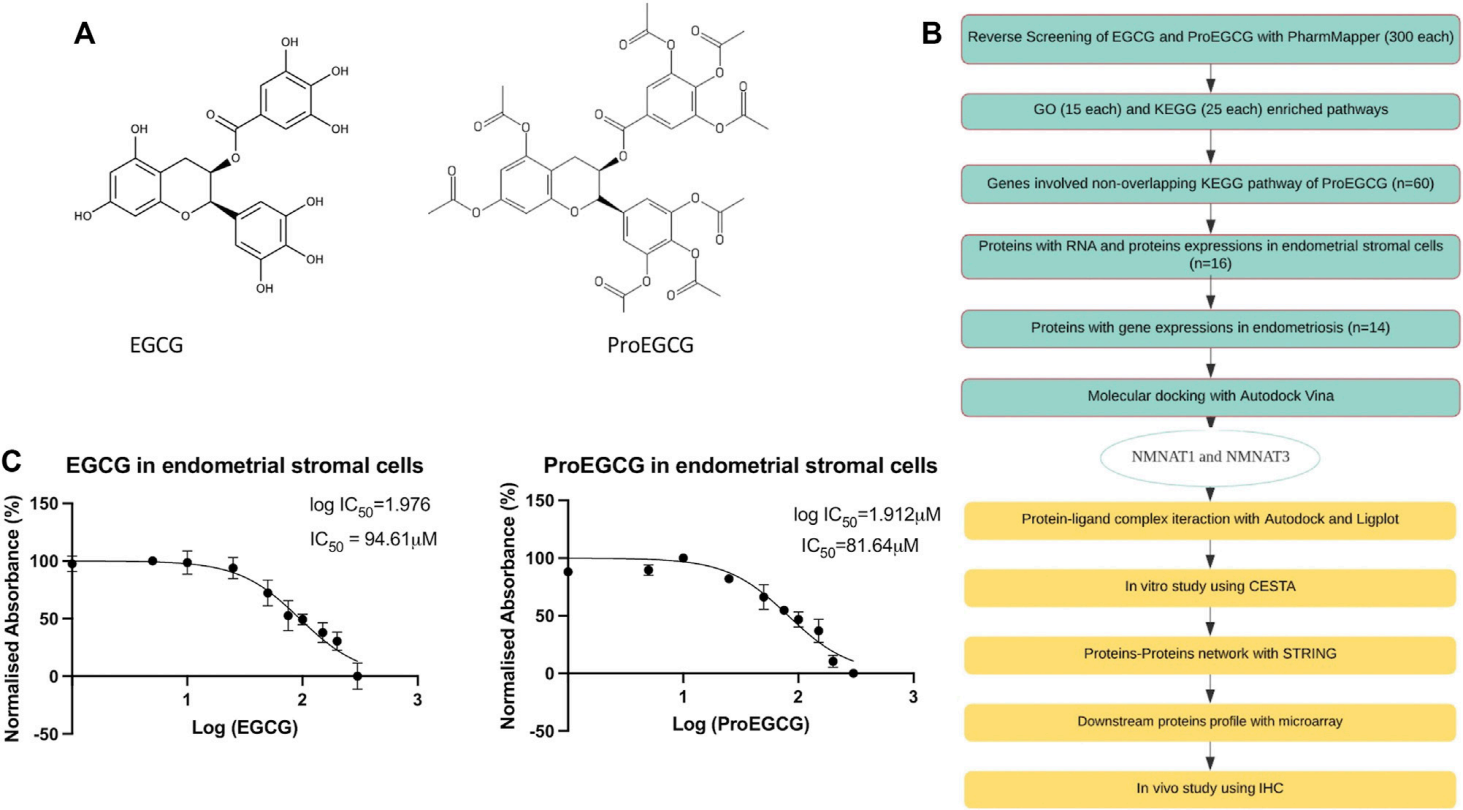
Study design. **(A)** Chemical structures of small molecules EGCG **(left)** and ProEGCG **(right)**. **(B)** Flowchart of methodology applied to identify and analyse the potential targets of EGCG and ProEGCG through various platforms. **(C)** MTT proliferation assays on human endometrial stromal cells after treatments of EGCG **(left)** and ProEGCG **(right)** in a range of 0–300 μM for 48 h. Normalised absorbance (%) vs. Log (drug concentrations) was plotted as mean ± SD (*n* = 3) and IC50 value was calculated usingGraphPad Prism version 8.4.2.

Epigallocatechin gallate (EGCG, Figure 1A) is a polyphenolic compound in green tea and is also a well-known antioxidant (Singh et al., 2011). A recent review article by Kamal et al. (2021) had showed promising effects of EGCG on female reproduction. The included studies in the review were conducted in animals and in human *in vitro* and *in vivo*. Previously, we reported its anti-endometriosis effect *in-vivo* (Xu et al., 2011; Xu et al., 2013). ProEGCG (Figure 1A), a prodrug of EGCG modified by Dou et al. (2008), also inhibited the developments of experimental endometriotic lesions in mice, with enhanced efficacy, bioavailability, anti-oxidation and anti-angiogenesis ability, compared to EGCG (Wang et al., 2013). However, the exact mechanisms of how EGCG and ProEGCG act as antioxidant agents and inhibit the lesions remain unclear. It is important to study the structure-activity relationship of EGCG and ProEGCG and identify their molecular and biological targets as the mechanism of action in treating endometriosis. This mechanistic information derived from drug-target interaction will guide further drug innovations (Santos et al., 2017). The pharmacological and physiochemical properties of EGCG and ProEGCG could be improved and be optimized into a new lead compound. Functional groups could be modified to minimize off-target events, drug toxicity, drug resistance, and to increase drug specificity and be developed into precision medicine.

This study aimed to identify molecular targets of ProEGCG in treating endometriosis, and to suggest a mechanism of action that explains the superior activities of ProEGCG. We employed a combined approach, starting with an *in-silico* study to find the potential proteins targets by reverse screening, followed by molecular docking and virtual screening of the complex structures. After that, we verified the *in-silico* binding results using cellular thermal shift assay *in vitro* to assess the stability of protein-small molecule complexes. Finally, we carried out an *in vivo* microarray with an experimental endometriosis mice model to study the molecular interaction of the selected targets after treatment.

## 2 Materials and Methods

### 2.1 *In Silico* Analysis

#### 2.1.1 Reverse Screening

PharmMapper (http://www.lilab-ecust.cn/pharmmapper/) was used to identify molecular targets based on pharmacophore mapping approach (Liu et al., 2010). The pharmacophore database contains 7,000 models by extracting targets from TargetBank, DrugBank, BindingDB and PDTD. 2D structures of EGCG and ProEGCG molecules in SDF format were submitted to the server and the best mapping poses of the molecules against all the pharmacophore models in the database were identified. Default settings were used for other parameters; the proteins species was limited to *Homo sapiens* only. The fit score of EGCG and ProEGCG to each pharmacophore were calculated and normalised to 0–1 range. The larger the fit score, the better the protein-molecule complex. Z-score was generated by combining the fit score to the database score matrix. It considered both pharmacophore models score, as well as the statistical factors such as the significance of the binding between protein targets to the compound. It compared the fit score of a specific pharmacophore to the scores level among all scores in the pharmacophore library. The larger the positive z-score, the more significance of the target to the compound and vice versa.

#### 2.1.2 KEGG and GO Enrichment Analysis

Top 300 potential protein targets of EGCG and ProEGCG, obtained from PharmMapper, were uploaded separately on the DAVID Bioinformatics Resources 6.8 server (https://david.ncifcrf.gov/summary.jsp) (Dennis et al., 2003) for GO functional and KEGG pathway enrichment analysis. The identifier was set to gene symbol, and *Homo sapiens* was selected to limit annotations in the gene list and background list. A significant value of *p* < 0.05 was set as the cutoff criterion. Common and different GO terms in biological process, cellular component, molecular function and top 25 enriched KEGG pathway were identified for further analysis.

#### 2.1.3 Endometrium and Endometriosis-Associated Targets Analysis

Proteins involved in the enriched KEGG pathways of ProEGCG were selected. RNA and protein expressions in endometrial stromal cells were analyzed for their ranges of RNA and protein expressions. Data were obtained from Human Protein Atlas Database (https://www.proteinatlas.org) (Uhlen et al., 2015; Thul et al., 2017). RNA and Protein expression levels were achieved by RNA-sequencing data and immunohistochemical staining patterns from internal, external sources and available protein and gene characterization public data. Gene expressions in endometriosis were obtained from Mammalian Gene expression database for uterus tissue (http://resource.ibab.ac.in/MGEx-Udb/) (Bajpai et al., 2012).

#### 2.1.4 Molecular Docking

Molecular docking was employed to predict the non-covalent binding of the target proteins to EGCG and ProEGCG. Autodock Tools, version 4.2 and Autodock Vina version 1.1.2 were used (Morris et al., 2009). The pdb structure of target proteins was downloaded from protein data bank (https://www.rcsb.org). The pdb structure of EGCG and ProEGCG were converted and exported from OpenBabel. Ligand binding sites of each protein were identified from metaPocket2.0 (Huang, 2009) (https://projects.biotec.tu-dresden.de/metapocket/index.php). Each ligand was docked individually to each protein. The grid box was set at a size of 20 × 20 × 20 points (x,y,z). It covered the binding site residues and allowed the ligand to move at a flexible condition. Grid spacing was set to 1 Å. Default settings were used for other parameters. The predicted ligand binding affinities were shown as a negative Gibbs free energy (kJ/mol). The more negative the binding affinity, the stronger the bonding of complex. Only proteins that show gene expression in endometrium, protein expression in the endometrial stromal cells, are expressed in endometriosis patients, and form protein-ligand complex with the most negative Gibbs free energy were selected as final potential protein targets for further chemical binding and functional analysis.

#### 2.1.5 Virtual Screening

Ligplot (https://www.ebi.ac.uk/thornton-srv/software/LIGPLOT/) (Wallace et al., 1995) was used for virtual screening after the molecular docking which gave a 2D interaction scheme. The software was used to observe the binding poses and to identify residues that involved hydrogen bonding and hydrophobic interaction in specific bonding distance as interaction radii in the complex. These were the non-covalent intermolecular interactions that influenced the binding affinity and the biological activity of drug.

#### 2.1.6 String Analysis

String version 11 (https://string-db.org) (Szklarczyk et al., 2019) was used for protein network analysis. NMNAT1 and NMNAT3 were submitted to the multiple protein search. Medium confidence with a threshold of 0.4 was used to define the protein-protein network. We also added a total of 10 proteins to show a network around the input by default enrichment. All active interaction sources were included based on the available evidence from literature, open databases and reported experiments. Enrichment analysis with the whole genome as a background dataset was used to identify the enriched terms and pathways.

### 2.2 *In Vitro* Analysis

#### 2.2.1 Chemicals

EGCG (Sigma Aldrich Cat. E4143, purity ≥ 95%) was purchased from Sigma. Prodrug of EGCG (ProEGCG) was a kind gift from Polytechnic University of Hong Kong and prepared according to literature procedure (Lam et al., 2004). ProEGCG (purity ≥ 99.8%, determined by LCMS/MS) is a peracetate derivative of EGCG, and DMSO was used as a solvent to dissolve EGCG and ProEGCG. The solution was diluted to the desired concentration with culture medium when used. Final concentration of DMSO was less than 0.1%.

#### 2.2.2 Cell Cultures

This study was conducted as approved by the Hospital Authority of Hong Kong and The Chinese University of Hong Kong, under ethics number CRE-9264. Primary human endometrial stromal cells from a healthy woman (*n* = 1) without endometriosis (hEsc) were obtained from endometrial biopsies for cell culture experiments. The patient gave informed consent for donating eutopic endometrial tissue for endometriosis research purposes. She was 42 years old, diagnosed with subserosal fibroids but without endometriosis, as confirmed by laparoscopy. The stromal cells were isolated by using collagenase Crude: type 1 (Sigma, cat. C2674) and purity was assessed by morphological determination. The Cell culture medium was DMEM/F-12 (Dulbecco’s Modified Eagle Medium/Nutrient Mixture F-12) (Thermo Fisher, cat. 21041025), with 10% FBS (Gibco, cat. 12676-029), 1% Amphotericin-B (Sigma, cat. A2942), 1% Pen-Strep (Gibco, cat. 1514066) and 1% L-Glutamines (Gibco, cat. 25030). Cells were passaged every 2 days at 80% of the confluency. RIPA buffer (Thermo Scientific, cat. 89900) with protease inhibitor (Thermo Scientific, cat. 78439) was used to lyse the cells for in-vitro assays. Protein concentrations were determined using DC™ Protein Assay (BioRad, cat. 5000112) with BSA as standard.

#### 2.2.3 MTT Proliferation Assay

hEsc was treated with EGCG and ProEGCG in a range of 0–300 μM for 48 h. Untreated cells were used as a negative control, while reagents with no cells was used as blank control. MTT (3-(4,5-Dimethylthiazol-2-yl)-2,5-Diphenyltetrazolium Bromide; ThermoFisher cat. M6494) was added to each well and incubated at 37°C for 3 h. DMSO was used as a solubilizing agent to dissolve the formazan crystals, and quantified with absorbance measurement at 570 nm. GraphPad Prism version 8.4.2. (https://www.graphpad.com/updates/). generated the MTT cell proliferation plot and calculated value of the IC50 concentration.

#### 2.2.4 Cellular Thermal Shift Assay

CESTA validated the protein-ligand binding without requiring small molecule modification, and solely depended on drug bindings, instead of drug actions (Jafari et al., 2014). Protein lysates of the cultured hEsc (2 μg/μl) were incubated with EGCG or ProEGCG at their IC50 concentrations or DMSO as vehicle control for 30 min at 25°C on a microplate incubator shaker. Complexes were heated for 3 min in seven different temperatures, ranging from 40°C to 70°C. Heat-induced unbound proteins to unfold while remaining soluble proteins were quantified. Soluble fraction of protein was isolated from the lysates by centrifugation for 15 min at 20,000 x g*.* Samples were then treated with SDS loading buffer for 10 min at 75°C, followed by western blotting analysis.

#### 2.2.5 Western Blotting

Denatured proteins were separated by 12.5% SDS/polyacrylamide gel electrophoresis (SDS-PAGE) and transferred to a PVDF membrane. The membrane was blocked with 5% skim milk in TBST for 30 min at room temperature. It was then probed with the corresponding primary antibodies, 1:200 Alpha tubulin (Santa cruz, cat. Sc-8035), 1:200 Beta actin (Santa cruz, cat. Sc-47778), 1:100 NMNAT3 (Santa cruz, cat. Sc-390433) and 1:100 NMNAT1 (Santa cruz, cat. Sc-271557) with gentle agitation overnight at 4°C. α-Tubulin was used as a positive control and ß-actin was used as a negative control protein (Chakrabarty et al., 2015). On the second day, the membrane was incubated with secondary antibodies, 1:500 Goat anti Mouse IgG1 HRP (Abcam, cat. A10551) with gentle agitation for 1 h at room temperature. The blots were then developed with ECL™ Prime Western Blotting Detection Reagent (GE Healthcare Amersham™, cat. 45-002-401). Western blot analysis were performed in triplicate. Protein bands were quantified by Fiji ImageJ software version 2.1.0/1.53c (https://imagej.net/software/fiji/). GraphPad Prism version 8.4.2. generated melting curves. The data were normalized in a range between 0 and 100, and was plotted as a sigmoidal curve of temperature vs percentage of unfolded protein.

### 2.3 *In Vivo* Analysis

#### 2.3.1 Experimental Endometriosis Model in Mice

All animal works were approved by the Animal Experimentation Ethics Committee of the Chinese University of Hong Kong and performed under ethics 07/010/MIS in accordance with institutional guidelines. 8-week-old C57BL/6 female mice provided by the Laboratory Animal Service Center were housed in a pathogen-free animal house with chow and tap water. Ovariectomy was performed 7 days before transplantation. 100 μg/kg estradiol-17β (E2; Sigma, Cat. E2758) were intramuscularly injected at the time of ovariectomy and on every 5 days to synchronize the E2 level. Mice in the donor group were sacrificed to obtain the endometriotic tissues of the uterine fragments, which was then cut into endometrial fragments of 2 mm in diameter using a dermal biopsy punch (Miltex). Endometriosis model was established on day 0 by subcutaneous transplantation. A 3 mm skin incision on the abdominal wall of each recipient mice was made, thus subcutaneous pockets were created. Three endometrial tissues were placed into the pocket and then the skin incision was closed with surgical thread (Wang et al., 2013; Xu et al., 2013). Anesthesia with 100 mg/kg ketamine, 10 mg/kg xylazine in 0.9% saline water was applied on all surgery. The mice were then randomly assigned to one of the following treatments: Vehicle as the negative control, EGCG and ProEGCG, injected intraperitoneally at 50 mg/kg every day for 21°days until termination for lesions collection and after mice were sacrificed. To assess the effects of EGCG and ProEGCG (*n* = 10 per treatment group) in inhibiting lesions growth, length, and width of the area of the endometriotic lesions were measured using a digital caliper. Lesions area (mm^2^) was determined using the formula, length (mm) × width (mm) × π/4.

#### 2.3.2 Microarray

Endometrial implants were collected in RNAlater solution at 4°C and incubated for 24 h, followed by removing the supernatant, and the tissues were stored at −80°C for long term storage. These were used for whole genome expression microarray analysis of mouse (*n* = 3 per treatment group). Total RNAs were extracted by the RNeasy mini kit (Qiagen) and were subjected to gene expression profiling using the Agilent microarray platform. For all RNAs, 260/280 ratio was ∼2, while RIN was ∼9. The experiment was performed according to previous procedures (Wang et al., 2013; Xu et al., 2013). Data were acquired by Feature Extraction 10.5 and were analyzed with GeneSpring GX 11.0 software (Agilent). T-test analysis was performed and *p* values of <0.05 were considered as significant. Multivariate variable pattern recognition of the target and related downstream proteins of NMNAT1 and NMNAT3 was performed. We transposed the dataset to have single animals as variable and gene expression as statistical units, plotted by Devtools and Ggbiplot from Bioconductor in R studio, with the animals on loading space (Gabriel, 1971; RStudioTeam, 2020). Differentially expressed genes were clustered using Cluster 3.0 (http://bonsai.hgc.jp/%7Emdehoon/software/cluster/) (de Hoon et al., 2004), and heatmap was generated and presented by Java Treeview (http://jtreeview.sourceforge.net) (Saldanha, 2004).

#### 2.3.3 Quantitative Real Time Polymerase Chain Reaction

To validate the microarray results and quantify gene expression levels of NMNAT1 and NMNAT3 in lesions of endometriosis mice model after EGCG or ProEGCG treatments, qRT-PCR was performed (*n* = 4–5 per treatment group). TB Green Premix Ex Taq (Tli RNaseH Plus) (Takara; cat RR420A) was used to amplify the gene expression and quantified by LightCycler 480 quantitative PCR system (Roche, Switzerland). Mouse GAPDH genes were used as house-keeping genes to normalize the gene expressions of studied proteins. PCR primers for NMNAT1, NMNAT3 and GAPDH were listed in Supplementary Table S1.

#### 2.3.4 Immunohistochemistry

Lesions tissues were fixed in 10% buffered formalin for 24 h and were embedded in paraffin blocks, cut into slices of 4 μm thickness. Sections were deparaffinized, incubated with 3% H_2_O_2_ in the dark to block the endogenous peroxidase activity. The sections were then incubated in boiled 10 mM sodium citrate buffer, pH 6 for antigen retrieval, and was boiled in a conventional microwave for 2 min for two times. It was then cooled and followed by blocking with 5% goat serum in 1% BSA/PBS and goat F(ab) anti-mouse IgG H&L (ab6668, 1 mg/ml, Abcam) to reduce non-specific signal and non-specific binding of the mouse serum to mouse tissue. The tissues were then incubated with primary antibodies 1:100 dilution of NMNAT3 (Santa cruz, cat. sc390433) and 1:100 dilution of NMNAT1 (Santa cruz, cat. sc271557) overnight, followed by secondary antibodies Goat Anti-Mouse IgG H&L (Abcam, cat. ab97023) at a dilution of 1:200 for 1 hour and developed in 3,3-diaminobenzidine (DAB) for 3–5 min, all in the appropriate dilution. After mounting, Leica DM6000B microscope was used for image capturing. For semi-quantitative analysis, NMNATs protein expression were quantified by H-score. The scores were generated blindly and independently by two authors (S. W. H and R. Z. Z). The H-score was calculated by summing up the percentage of different intensity level-stained cells: strongly stained cells (3+), moderately stained cells (2+), weakly stained cells (1+) and unstained cells (0) of a fixed field, on a continuous scale of 0–300, H-score = [1 × (% 1+) + 2 × (% 2+) + 3 × (% 3+)] (Fedchenko and Reifenrath, 2014; Parris et al., 2014). Each field was selected randomly and was repeated to capture five fields. The final cell count was reported as average of the five fields, and H-score was reported as whisker plot with median shown.

#### 2.3.5 Statistical Analysis

In the animal experiments, between groups comparisons were made using one-way ANOVA analysis. Tukey post-hoc test was used for multiple comparisons. Statistical analyses were performed by Graphpad Prism. *p*-value of ≤0.05 was considered statistically significant.

## 3 Results

The chemical structures of EGCG and ProEGCG were submitted to PharmMapper, and the top 300 best-fitted protein targets were obtained (Supplementary Table S2). Flow of the study protocol was presented as in Figure 1B. MTT proliferation assays identified the IC_50_ values of EGCG and ProEGCG in the endometrial stromal cell, which were 94.6 and 81.6 μM (Figure 1C).

### 3.1 Identification of the Differential Mechanism and Targets of EGCG and ProEGCG

**FIGURE 2:**
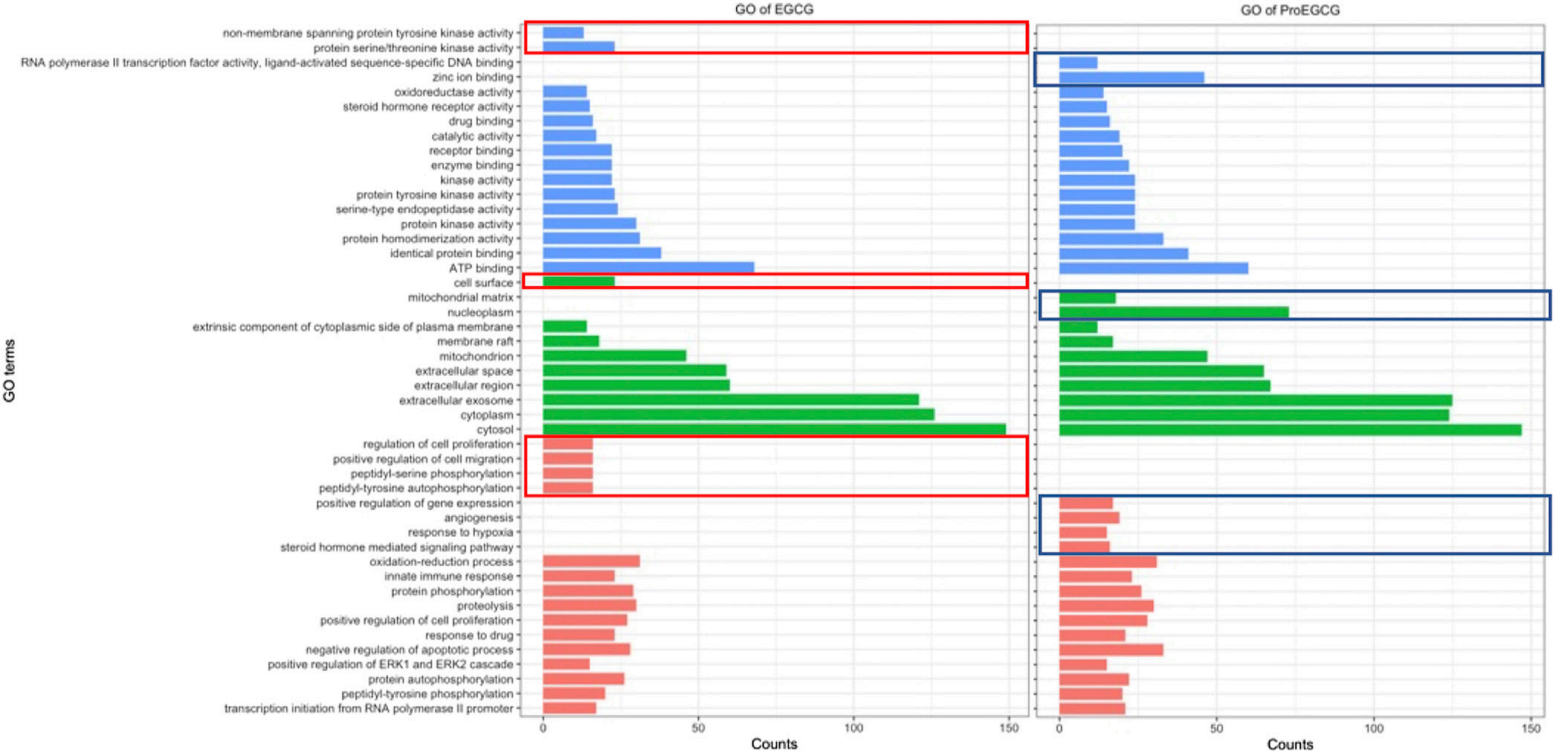
GO functions EGCG **(left)** and ProEGCG **(right)**. Differential functions of EGCG and ProEGCG were highlighted in red (EGCG) or blue (ProEGCG).

**FIGURE 3:**
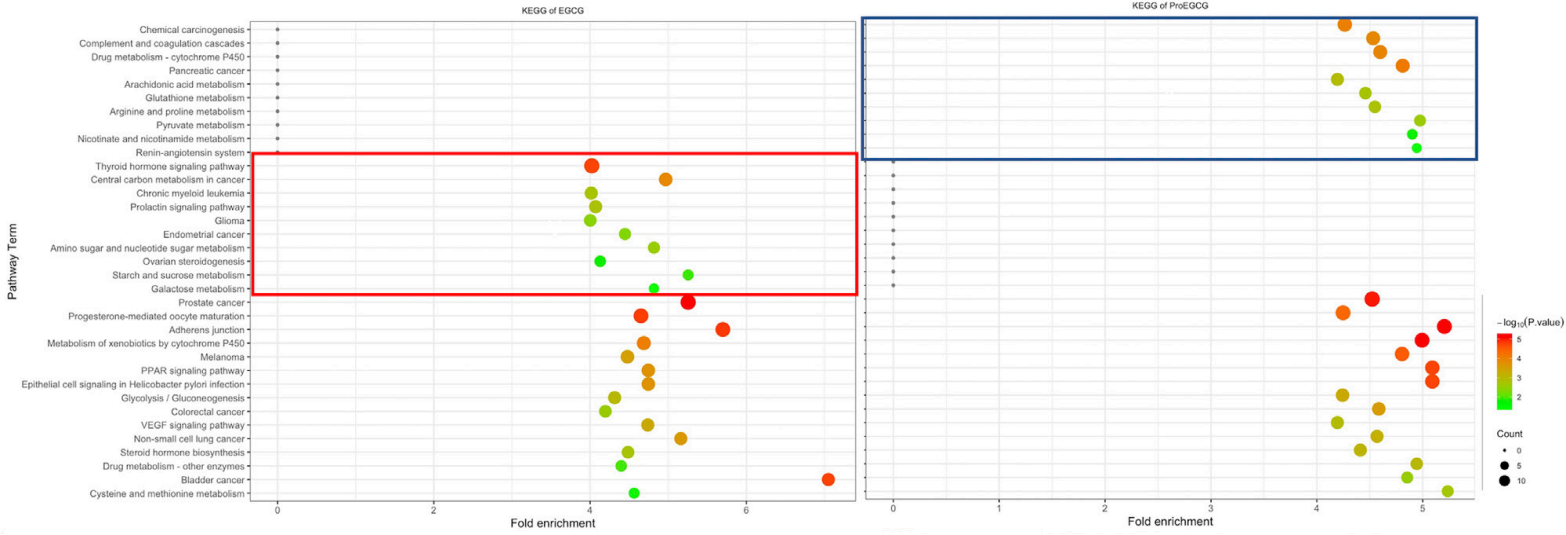
Differentia KEGG pathway of EGCG **(left)** and ProEGCG **(right)**. Differential pathways of EGCG and ProEGCG were highlighted in red (EGCG) or blue (ProEGCG).

To identify and differentiate mechanism of actions of EGCG and ProEGCG, we performed GO and KEGG enrichment analysis of EGCG and ProEGCG with each of the 300 proteins targets separately by using software platform DAVID (Dennis et al., 2003). The top 15 GO functions of each term (Figure 2) and the top 25 enriched KEGG pathways of EGCG and ProEGCG were shown (Figure 3; Supplementary Table S3). Terms of GO and KEGG from both groups were aligned and enriched terms from either ECGC or ProEGCG were shown in Figures 2, 3 and Supplementary Data Sheet S1. EGCG was differentially participated in protein serine/threonine kinase activity, regulation of cell proliferation, migration, peptidyl-serine phosphorylation, and peptidyl-tyrosine autophosphorylation; and was involved in endometrial cancer, as well as a range of metabolic pathways that included: starch and sucrose metabolism, central carbon metabolism in cancer, galactose metabolism; and amino sugar and nucleotide sugar metabolism. ProEGCG differentially participated in RNA polymerase II transcription factor activity and regulation of gene expression, angiogenesis, responses to hypoxia, and steroid hormone-mediated signaling pathway; and was involved in renin-angiotensin system, as well as a range of metabolic pathways that included: pyruvate metabolism, nicotinate and nicotinamide metabolism, drug metabolism in cytochrome P450, arginine and proline metabolism; and glutathione and arachidonic acid metabolism.

To study the differentiated proteins targets of ProEGCG over EGCG and relate the targets with its selective mechanism of action, a total of 60 proteins from the top 10 enriched pathways of ProEGCG, as mentioned above, were selected for further analysis (Supplementary Table S4). Datasets of expressed RNA, proteins in endometrial stromal cells and gene status in human endometriosis patients of the potential target proteins were extracted. 44 proteins that had no protein expression in endometrial stromal cells were excluded, and two proteins with no gene expression in endometriosis patients were excluded. 14 proteins were included for molecular docking. Gibbs free energy of ProEGCG-protein complexes was calculated. NMNAT1 and NMNAT3 had expressions in the endometrial stromal cells (Uhlen et al., 2015; Thul et al., 2017), were genes that undergo transcription in endometriosis (Bajpai et al., 2012) and had the most negative Gibbs free energy, denoting the most substantial binding with ProEGCG (NMNAT1: −8.7 kJ/mol; NMNAT3: −10 kJ/mol).

### 3.2 Validation of the Proteins’ Targets With Cellular Thermal Shift Assay

**FIGURE 4:**
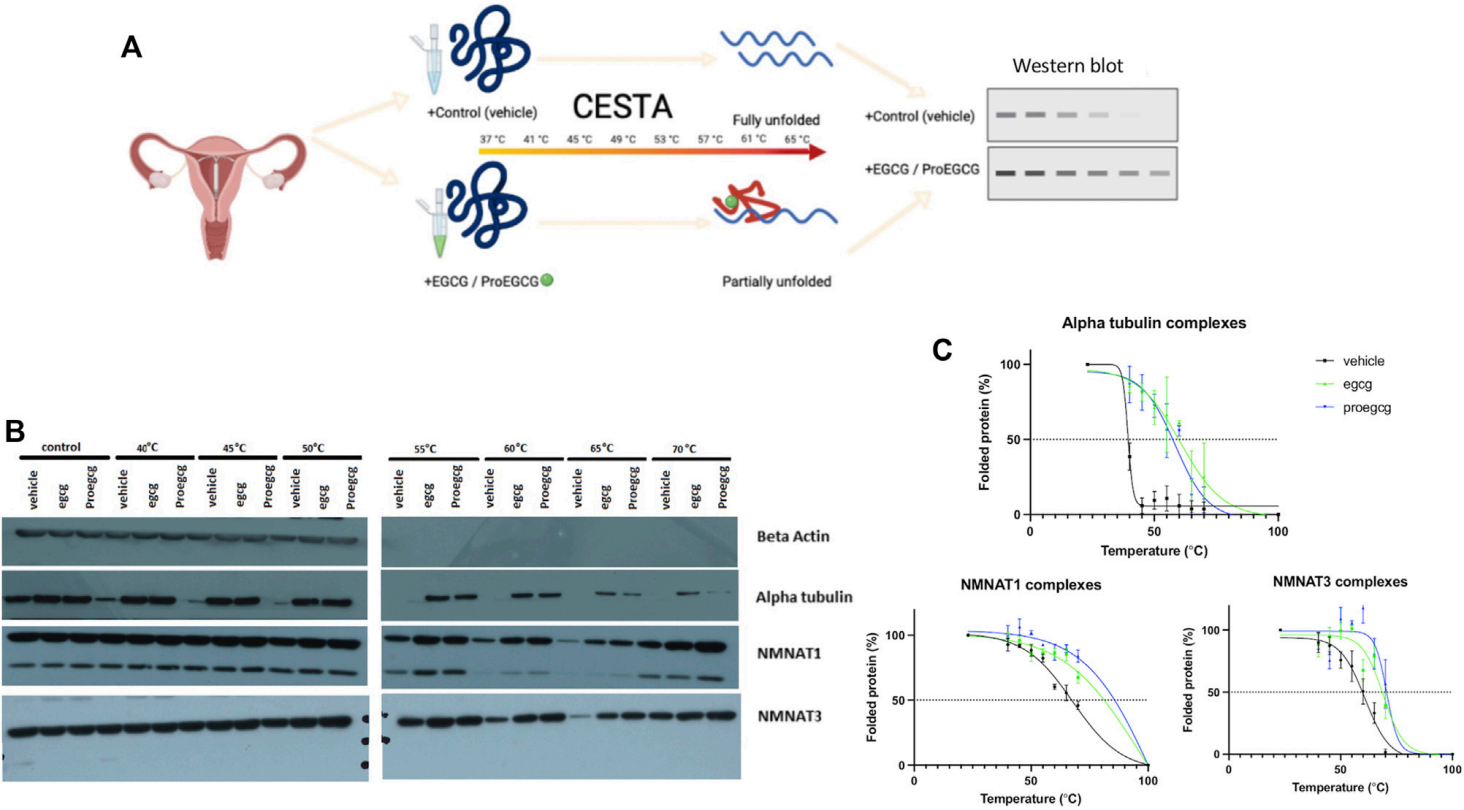
CESTA *in vitro* bindling assays. **(A)** Schematic diagram showing the *in vitro* binding assay, Cellular Thermal Shift Assay (CESTA), to verify direct protein targets of EGCG and ProEGCG. Target proteins of drugs are resistance to thermal heating due to protein structure was stabilized upon drug binding, thus proteins will be partially unfolded; that without drug binding will be fully unfolded. **(B)** Western blot analysis was performed in triplicates for CESTA, representative western blot shows stronger intensities in drug binding targets proteins than vehicle, upon thermal heating. **(C)** Melting curves of CESTA. Relative band intensities were plotted against corresponding incubation temperatures of each protein ligand complexes, as mean ± SD (*n* = 3). Boltzmann sigmoidal fit was applied. Melting curves were generated by GraphPad Prism. NMNAT1: Nicotinamide Nucleotide Adenylytransferase 1; NMNAT3: Nicotinamide Nucleotide Adenylytransferase 3.

CETSA assay was used to validate the protein-ligand complex stability upon thermal treatment (Figure 4A), shown by western blot, and analysed through melting curves plottings. NMNAT1 and NMNAT3 as likely protein targets of ProEGCG were proceeded for validation (Figure 4B). Melting temperatures of complexes were compared (Figure 4C), and obtained when 50% of proteins were unfolded. NMNAT1-ProEGCG (85°C) and NMNAT3-ProEGCG (75°C) had a higher melting temperature than the NMNAT1-vehicle (65°C) and NMNAT3-vehicle (60°C) as well as NMNAT1-EGCG (80°C) and NMNAT3-EGCG (70°C) respectively. Alpha tubulin-EGCG (65°C) had a higher melting temperature than the Alpha tubulin-vehicle (48°C) and Alpha tubulin-ProEGCG (58°C). CESTA supported the differentiated affinities energy calculated by Autodock.

### 3.3 NMNAT1 and NMNAT3 as Potential Targets of ProEGCG Over EGCG

**FIGURE 5:**
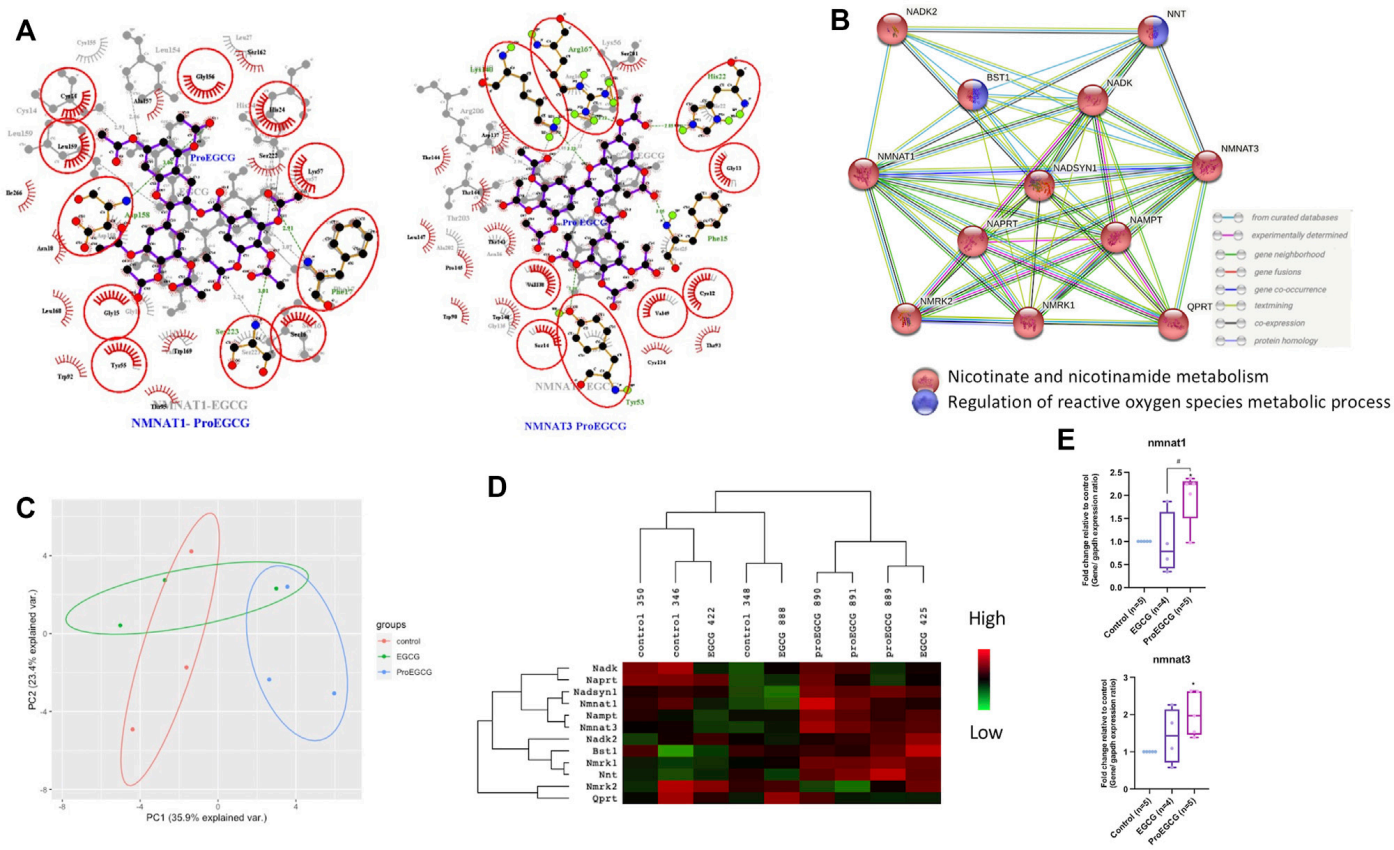
Network analysis. **(A)** ligplot shows the interaction analysis of structures of protein-ligands binding sites in a 2D interaction scheme are shown. Overlap of two complexes NMNAT1-Ligands (left) and NMNAT3-Ligands (right) as shown. Residues that are involved in both complexes are denoted in red circles. Pro EGCG complexes, labelled in blue colour are on top and EGCG complexes, labelled in grey colour are on bottom of each figure. **(B)** Protein-Protein interaction network by STRING showing interaction of NMNTA1 and NMNAT3 with other proteins. **(C)** Principal component analysis score plot with the microarray expression profiles in different treatments (*n* = 3). **(D)** Heatmap shows the downstream proteins expression levels in different treatments of endometriosis mice model in microarray analysis (*n* = 3). Heatmap was generated and presented by java Treeview. Rows and columns are mean-centered, with relative abundance represented by color (green, lower abundance; red, higher abundance). **(E)** qRT-PCR analysis was conducted by measuring the fold change of gene expression levels in lesions of endometriosis mice model after EGCG or ProEGCG treatments. Gene levels were normalized relative to control and GAPDH as housekeeping gene. The fold change between groups were compared by whisker plot (*n* = 4–5). Top and bottom box edges represented the third and first quartile. The whiskers represented highest and lowest score. Their median were shown as the vertical line inside the box. One way ANOVA with Tukey post-hoc test was used for the multiple group’s comparison. *p < 0.05, compared to control; ^#^ *p* < 0.05 compared to only EGCG/ProEGCG.

2D interaction schemes of the NMNATs-ligand complexes were shown (Figure 5A). The hydroxyl groups of EGCG contributed to the hydrophilicity property and were recognized as a hydrogen bond donor or accepter. The acetyl groups of ProEGCG contributed to the hydrophobicity property interacted with amino acid that has a hydrophobic side chain such as alanine, leucine, and valine to stabilize the complexes. String retrieved the interacting genes of NMNAT1 and NMNAT3 in a protein–protein interaction (PPI) networks (Figure 5B) (Szklarczyk et al., 2019). Ten interacted proteins were enriched and were all involved in nicotinate and nicotinamide metabolism. BST1 and NNT were involved in the regulation of reactive oxygen species metabolic process.

### 3.4 ProEGCG Upregulates NMNAT1 and NMNAT3 Expression Levels in Endometriosis Mice Model

**FIGURE 6:**
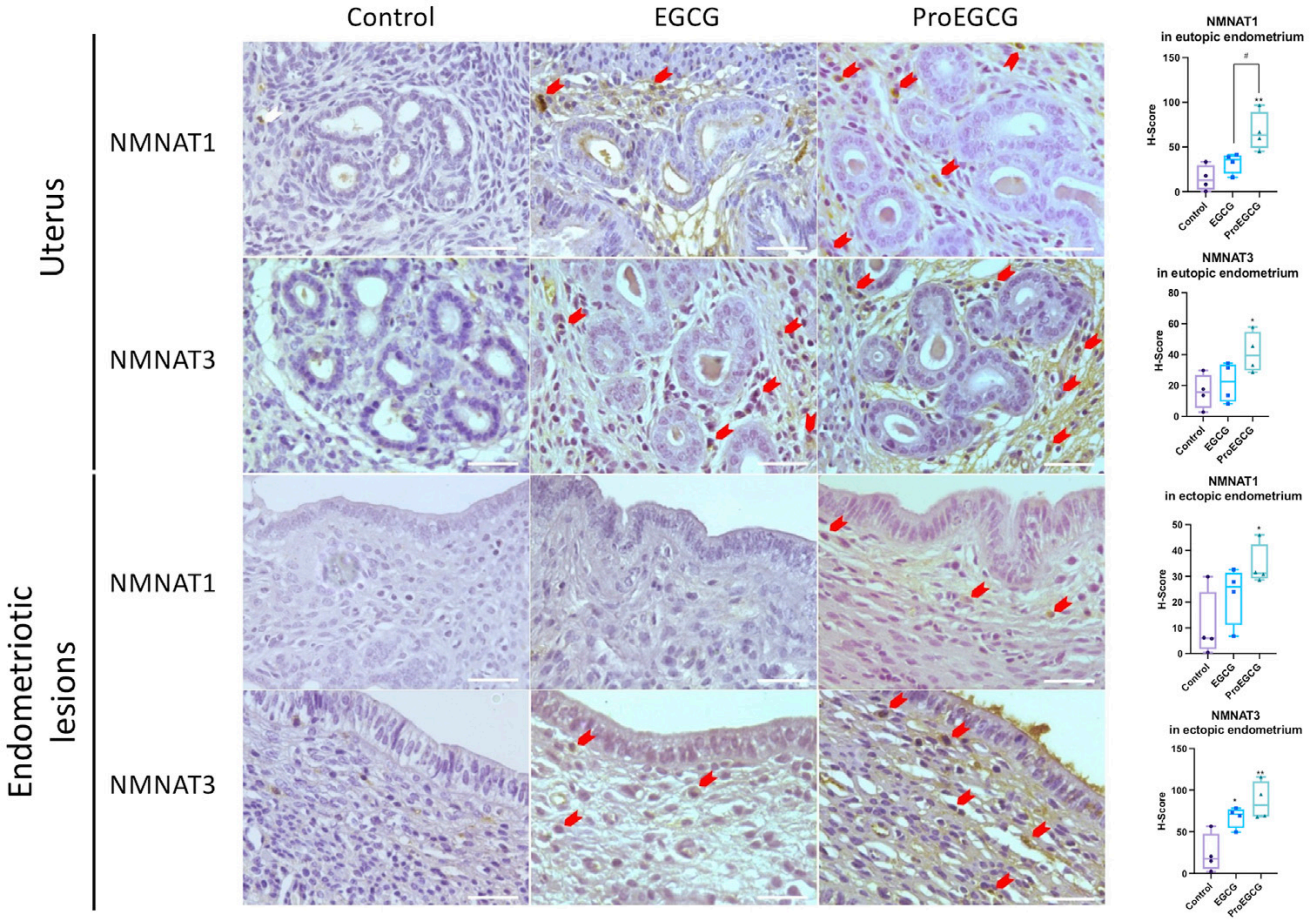
Protein expressions of NMNAT1 and NMNAT3 in uterus and endometriotic lesions of EGCG and ProEGCG- treated and untreated groups, determined by immunohistochemistry. Representative images in different groups are shown, red arrow denoted positives staining cells. The H-score for NMNATs expression in mice eutopic and ectopic endometrial tissues were compared by whisker plot (*n* = 4). Top and bottom box edges represented the third and first quartile. The whisker represented highest and lowest score. There median were shown as the verticle line inside the box. One-way ANOVA with Tukey post-hoc test was used for the multiple group’s comparison. *p < 0.05, **p < 0.01, compared to control ^#^ *p* < 0.05 compared to only EGCG/ProEGCG. Scale bar = 50 µm.

Endometriosis mice model was successfully developed to study the therapeutics effects of EGCG and ProEGCG, and the interaction involved in NMNAT1 and NMNAT3 PPI network, as enriched by Strings. Lesions sizes were significantly reduced in EGCG and ProEGCG groups, compared to the control (Supplementary Figure S1). The inhibitory effect from ProEGCG on lesions growth was more significant than that from EGCG. Microarray studied the gene expressions of downstream proteins. Nmnat1, Nmnat3 and downstream targets did not reach a fold change >2 (Supplementary Data Sheet S2), however the PCA model of the data set from the three groups captured 59.3% of the total variance of data, which includes 35.9% of principal component 1 and 23.4% of principal component 2 (Figure 5C) (Gabriel, 1971; RStudioTeam, 2020). PCA model showed that ProEGCG group was scattered on the right, while control group was scattered on the left. A heatmap based on hierarchical clustering of both rows and columns was built using an average linkage clustering algorithm (Figure 5D). Nadsyn1, Nampt, as well as Nmnat1 and Nmnat3 were upregulated in the ProEGCG group, while Qprt, Nmrk2, Naprt and Nadk were downregulated in ProEGCG group. To validate the result from microarray analysis, gene expression levels of Nmnat1 and Nmnat3 were analyzed by qRT-PCR (Figure 5E). ProEGCG significantly upregulated gene expression levels of Nmnat1 and Nmnat3. In addition, immunohistochemistry showed that ProEGCG significantly upregulated the proteins expression of Nmnat1 and Nmnat3 in stromal cells of endometriotic lesions and uterus after treatments, while EGCG only significantly upregulated that of Nmnat3 expression in lesions (Figure 6; Supplementary Figure S2).

## 4 Discussion

Green tea is a commonly consumed beverage worldwide, which is proven to have many health benefits from immunity strengthening to cancer prevention (Suzuki et al., 2012). Green tea contains many polyphenolic flavonoid-derived compounds known as catechins, and EGCG is the most abundant and active antioxidant among polyphenolic catechins (Zaveri, 2006). The presence of the gallate moiety on EGCG enhances the anticancer property of catechins (Saeki et al., 2018). An extra phenol ring on EGCG improves its antioxidant property. As a scavenger of free radicals, it traps electrons represses ROS activity and reduces oxidative stress (Bernatoniene and Kopustinskiene, 2018). Many other studies showed that EGCG increases DNA repair, apoptosis, immune system, and reduces inflammation and angiogenesis (Singh et al., 2011; Chu et al., 2017). Dou et al. showed a novel prodrug of EGCG (ProEGCG), which improved the stability and bioavailability of EGCG under physiological conditions. This is done by introducing the acetyl groups on the hydroxyl groups of EGCG (Dou et al., 2008) which prevented the production of reactive phenoxide anions and radicals, as well as dimerization (Chen et al., 2001). Both EGCG and ProEGCG were shown as potential anti-oxidation and anti-angiogenesis agents for endometriosis (Xu et al., 2011; Wang et al., 2013; Xu et al., 2013). However, bioavailability, oxygen radical absorbance capacity in plasma, and apoptosis in endometriotic lesions were significantly higher in ProEGCG, while level of VEGF concentration in plasma was significantly lower after ProEGCG treatment parameters were compared with control and EGCG groups (Wang et al., 2013). Therefore, our current study aimed to explore the potential molecular binding targets of ProEGCG over EGCG in increasing antioxidant capacity for endometrial tissues and endometriosis lesions *via in silico* reserve screening approach, followed by *in vitro* validation and *in vivo* assays to study the genes and protein expression. This is the first study that identified NMNAT1 and NMNAT3 as protein targets of ProEGCG *in silico* and *in vitro*, which are overexpressed after ProEGCG treatment *in vivo*. The stronger anti-oxidation effect of ProEGCG over EGCG could be explained by activating NMNATs.

*In silico* molecular study determines the targets solely based on chemical structures and without the involvement of metabolism actions. In general, a prodrug is metabolically or physiochemically converted into a pharmacologically active agent (Zawilska et al., 2013). It might bind to the same targets, leading to similar activities. However, our *in silico* molecular study suggested that differences in the chemical structures and functional groups of EGCG and ProEGCG lead to different involvement of intermolecular interactions, resulting in different affinity energies. We investigated the targets by differentiating the enriched pathways. Although EGCG and ProEGCG had similar anti-endometriosis activities, their different structures suggested that they might bind to different targets which implied a different mechanism of action. In our study, NMNAT1 and NMNAT3 were potential protein targets of ProEGCG. CESTA verified the accuracy of calculated binding affinities from computational results. The *in vitro* study validated the results from *in silico*. Upon drug binding on the target proteins, the proteins exerted extra stability and resistance against thermal heating, while unbound proteins would be unfolded and precipitated. NMNAT1-ProEGCG and NMNAT3-ProEGCG resulted in a higher melting point than corresponding pairs of another ligand.

NAD^+^ and its reduced form, NADH, maintain the level of NAD^+^/NADH, in the tricarboxylic acid (TCA) cycle and play a role in intracellular redox and metabolic state in cells. NMNATs are enzymes that are involved in the biosynthetic process to convert nicotinamide mononucleotide (NMN) and adenosine triphosphate (ATP) to NAD^+^ (Jayaram et al., 2011; Wilhelm and Hirrlinger, 2011). However, they do not consume NADH and for conversion to NADPH, which is a cofactor of ROS-scavenging enzymes (Mazzio and Soliman, 2003; Press and Milbrandt, 2008). Degradation and biosynthesis of NAD^+^ maintain cellular homeostasis. Low NAD^+^ and low NAD^+^/NADH ratio could lead to mitochondrial dysfunction and metabolic disease (Srivastava, 2016; Goody and Henry, 2018). Mitochondria participates in ATP production, and in ROS production (Ryan and Hoogenraad, 2007; Bratic and Larsson, 2013). Dysfunctional mitochondria elevate ROS production indirectly (Murphy, 2013). Therefore NAD^+^ is important in regulating ROS in mitochondria (Murphy, 2009). NAD^+^ was also shown to attenuate oxidative DNA damage due to exposure of amyloid beta-peptide in cortical neurons (Wu et al., 2014), and activated autophagy to reduce oxidative stress in retinal pigment epithelium cells *in vitro* (Zhu et al., 2016). It was thus proposed as a novel agent to treat oxidative stress-related disease (Zhu et al., 2016).

NMNAT3 is localized in mitochondria and can regulate mitochondrial metabolism. Previous studies showed that overexpression of NMNAT3 increased NAD^+^ levels in various tissues in mice and subsequently promoted mitochondrial respiration with a higher level of ATP and lower level of ROS (Gulshan et al., 2018). Another study also showed that NMNAT3 mediated protection against ROS damage on axonal degeneration during mitochondrial injury (Press and Milbrandt, 2008). On the other hand, NMNAT1 is localized in nuclei and increases NAD^+^, as a substrate for sirtuin (SIRT) consumption. SIRT had been widely studied for its protective effects against oxidative stress (Rajendran et al., 2011; Singh et al., 2018). Women with endometriosis were found to have a low level of ATP, and ATP linked mitochondrial respiration level (Hsu et al., 2015; Horne et al., 2019), implying mitochondrial dysfunction effect of these in regulating ROS was never studied in eutopic or ectopic endometrium. Resveratrol, a polyphenol and a potentially effective therapeutics option for endometriosis, was found as a SIRT1 activator through upregulating NMNAT1 to increase NAD^+^ (Grant, 2010; Taguchi et al., 2014). Nevertheless, SIRT1 suppressed inflammatory cytokines in endometriosis (Taguchi et al., 2014), and activated NF-kB deacetylation to induce apoptosis in tumors (Chao et al., 2017).

Target proteins do not always induce expression changes, and proteins with expression level changes are not always the targets of small molecules. However, if a protein binds to the small molecules and induces an expression change, it has excellent potential as a target protein. *In vivo* results confirmed that NMNAT1 and NMNAT3 were upregulated in both RNA and protein levels after treatment of ProEGCG. Differential action of mechanism of ProEGCG depends on its binding to NMNAT1 and NMNAT3, as well as the strength of induction. Although the exact mechanism of NMNATs in regulating redox reaction and oxidative stress in endometriosis remains unknown, the role of NMNATs in regulating NAD^+^, which plays an essential role as a redox carrier in cells is widely studied. There were some limitations of current studies. KEGG enriched genes in nicotinate and nicotinamide metabolism pathway included NMNAT1, NMNAT3, NT5M, BST1 and PNP. NMNAT1 and NMNAT3 are expressed in endometrial stromal cells, but PNP, NT5M and BST1 are expressed in endometrial epithelial cells only. Endometrial stromal and epithelial cells dysregulate different molecular pathways and have different molecular effects on endometriosis patients, yet the cells have interacting functions. The endometrial stromal cell is responsible for epithelial cells development and serves as an inducing factor for the growth of endometrial nodules (Mai et al., 1997). Our study identified dysregulated targets in stromal cells and provided a cell-type specific approach to treat endometriosis, as suggested by Logan et al. (2018). The protein targets of epithelial cells and their effects on endometriosis require further study. Another limitation was that reverse screening *in silico* could not screen endometriotic disease-specific targets of EGCG and ProEGCG. We therefore filtered off-targets that had no expression in endometrium and had no gene expression in endometriosis patients. Meanwhile, our study relied on CESTA to validate the *in silico* reverse screening. Chemical proteomics with mass spectroscopy could be an extra and better approach to identify drug molecular targets in a high throughput screening scale (Jafari et al., 2014; Gaetani et al., 2019). Although our current study was conducted with relatively small sample size, the therapeutic effects, as previously reported and the results of *in vivo* qPCR and immunohistochemistry work were still significant, with statistical power >90% and confidence interval within range across all dataset. The therapeutic effects of ProEGCG were not studied in human samples. In the present study, we did not study and compare the downstream functional and mechanistic effects of ProEGCG with EGCG, which was already shown in our previous publication. IC50 values of EGCG and ProEGCG were not dramatically different as these were obtained from whole cell experiments. We did not justify the subsequent analysis on different mechanism of actions by this approach. Instead, we incubated the protein lysates of the cultured hEsc with EGCG or ProEGCG at their IC50 concentrations for CESTA analysis to validate the *in silico* screening. This indicated the differential binding of NMNAT1 and NMNAT3 to EGCG and ProEGCG. The present study employed a combined approach to identify the potential binding targets of ProEGCG to treat endometriosis.

## 5 Conclusion

Our study suggested that ProEGCG binds to NMNAT1 and NMNAT3 molecules in human endometrial stromal cells and validated in endometriosis mouse model. We reported for the first time a combined approach to identify the potential targets and pathway of ProEGCG with superior effects over EGCG potentially for endometriosis treatment. The binding of ProEGCG to NMNATs could potentially scavenge ROS and inhibits the progression of endometriosis. Future works may investigate the role of NMNATs in regulating oxidative stress and the mechanism of actions by ProEGCG targeting NMNATs binding, particularly their anti-oxidation effects in treating endometriosis.

## Data Availability

The original contributions presented in the study are included in the article/Supplementary Material, further inquiries can be directed to the corresponding authors.
