# Supplementary figures and images for: An In-Silico, In-Vitro and In-Vivo Combined Approach to Identify NMNATs as Potential Protein Targets of ProEGCG for Treatment of Endometriosis
Source: Front Pharmacol. 2021 Oct 14;12:714790. doi: 10.3389/fphar.2021.714790 (PMC8552031; doi:10.3389/fphar.2021.714790)

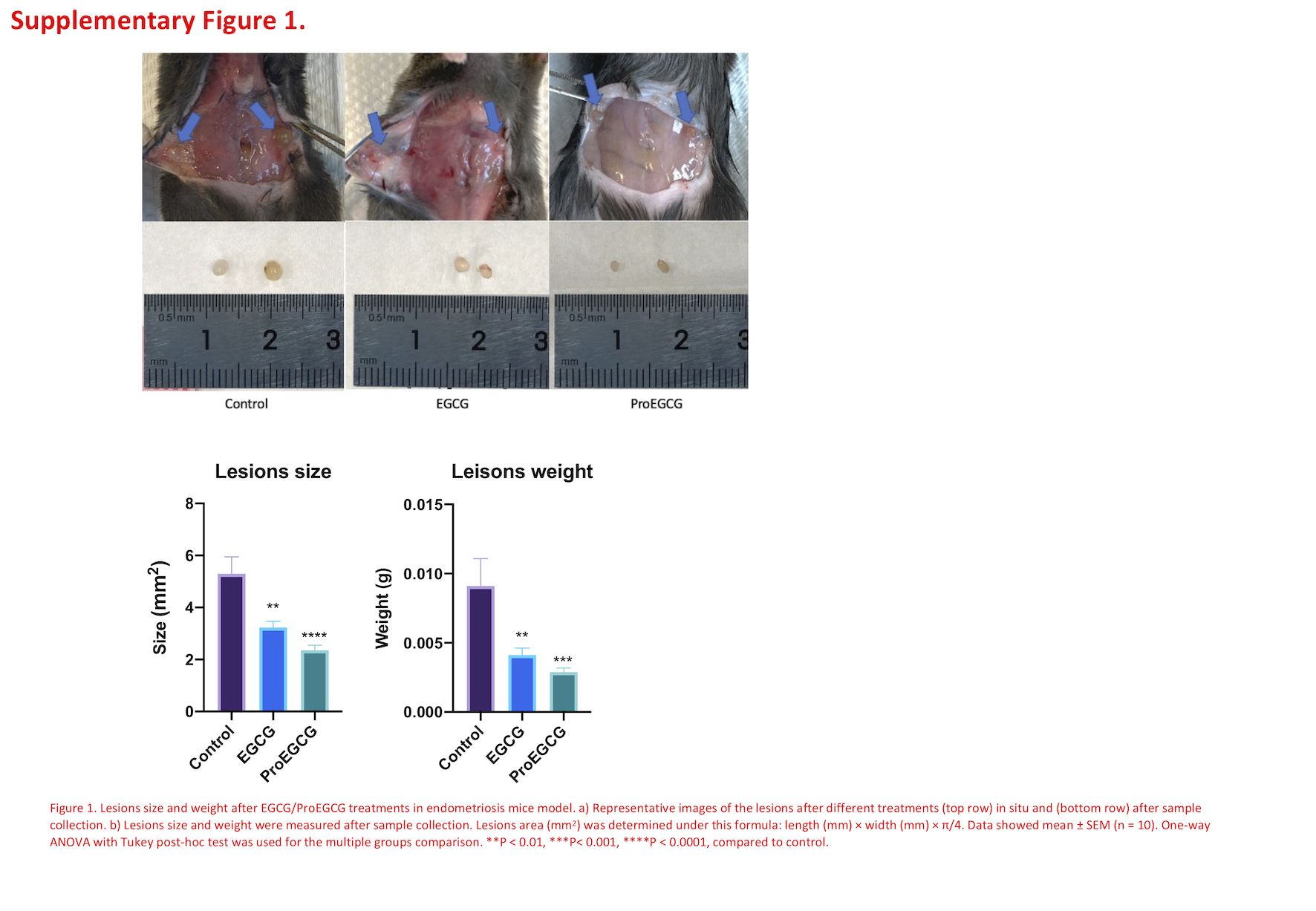

Supplement: Supplementary file 2 [file Image1.JPEG]

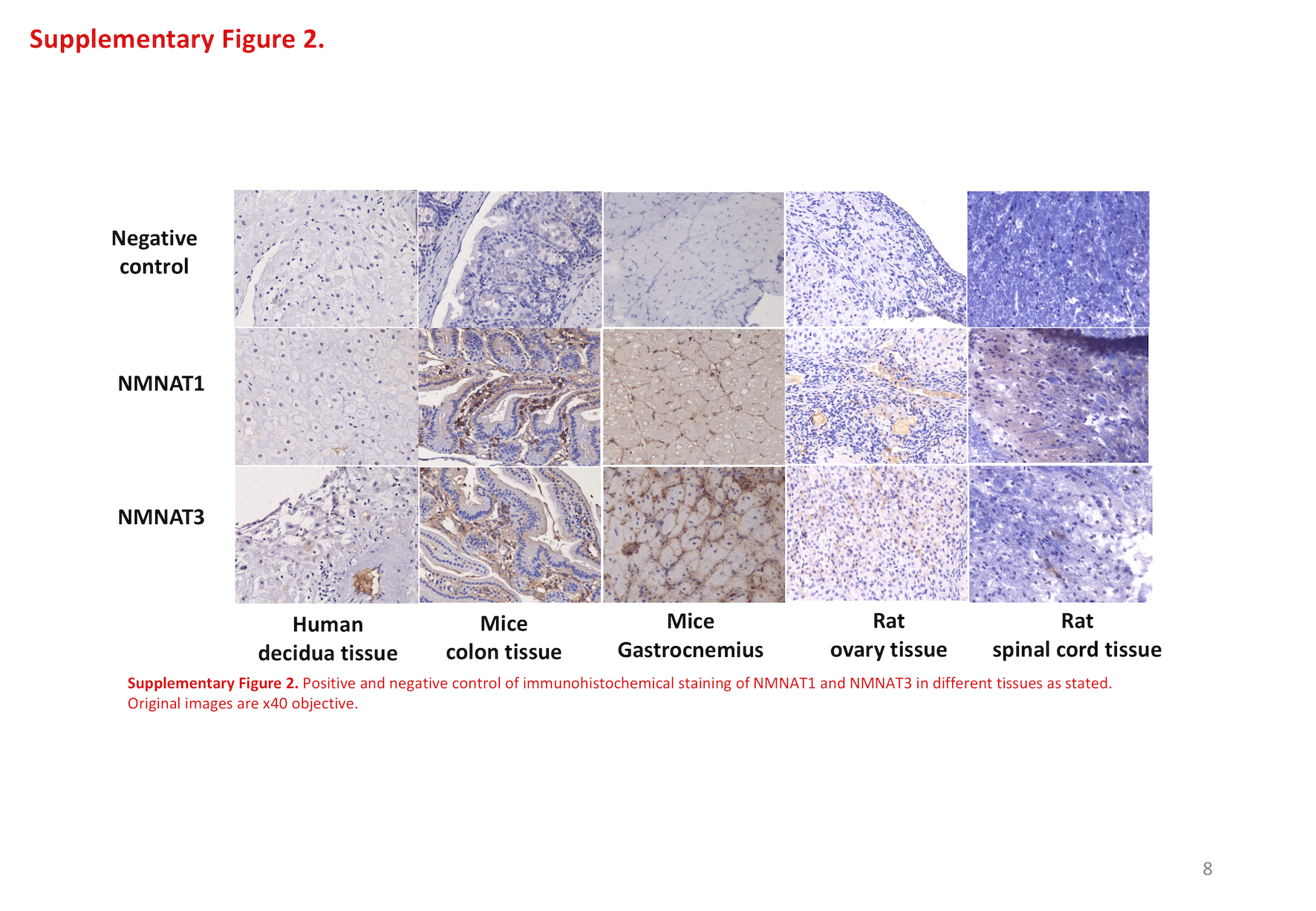

Supplement: Supplementary file 3 [file Image2.JPEG]
