# Supplementary material for: An In-Silico, In-Vitro and In-Vivo Combined Approach to Identify NMNATs as Potential Protein Targets of ProEGCG for Treatment of Endometriosis
Source: Front Pharmacol. 2021 Oct 14;12:714790. doi: 10.3389/fphar.2021.714790 (PMC8552031; doi:10.3389/fphar.2021.714790)
Supplement: Supplementary file 4 [file DataSheet1.pdf]

Supplementary Table 1.

Gene	Sequences
nmnat1 Forward Primer	TGGCTCTTTTAACCCCATCAC
nmnat1 Reverse Primer	TCTTCTTGTACGCATCACCGA
nmnat3 Forward Primer	ATCACGAATATGCACCTGCG
nmnat3 Reverse Primer	ATTGACGGGTGAGATGATGCC
gapdh Forward Primer	AGGTCGGTGTGAACGGATTTG
gapdh Reverse Primer	TGTAGACCATGTAGTTGAGGTCA

Supplementary Table 1. Primer sequences for quantitative real-time PCR.

Supplementary Table 3.

KEGG terms	ProEGCG: Fold Enrichment	ProEGCG: P value	ProEGCG: Count	EGCG: Fold Enrichment	EGCG: P value	EGCG: Count
Prostate cancer	4.522257701	0.00001	14	5.255156608	2.47604E-06	15
Metabolism of xenobiotics by cytochrome P450	4.993689971	0.00000829	13	4.687031569	3.94965E-05	12
Adherens junction	5.204690956	0.00000531	13	5.69925435	6.81546E-06	14
Progesterone-mediated oocyte maturation	4.247506412	0.0000447	13	4.651115619	7.34864E-06	14
Epithelial cell signaling in Helicobacter pylori infection	5.091155791	0.0000176	12	4.745327982	8.59112E-05	11
PPAR signaling pathway	5.091155791	0.0000176	12	4.745327982	8.59112E-05	11
Melanoma	4.804330113	0.000031	12	4.47798556	0.000141754	11
Colorectal cancer	4.584777393	0.000274	10	4.195649227	0.001193955	9
Glycolysis / Gluconeogenesis	4.242629826	0.000496	10	4.313934529	0.000438899	10
Steroid hormone biosynthesis	4.410872043	0.00085	9	4.485004347	0.00076191	9
Non-small cell lung cancer	4.568403188	0.000669	9	5.161314526	0.000108128	10
VEGF signaling pathway	4.19394391	0.001193499	9	4.738255958	0.000213044	10
Drug metabolism - other enzymes	4.943586058	0.000988	8	4.398337596	0.004632482	7
Cysteine and methionine metabolism	5.236298391	0.001869921	7	4.563688633	0.0092286	6
Bladder cancer	4.853154606	0.002790321	7	7.049600328	7.85009E-06	10

Supplementary table 3. Top 15 enriched common KEGG pathways of EGCG and ProEGCG, along with Fold enrichment values, P values and genes count.

Supplementary Table 4.

PDB	Gene symbol	Target name	RNA expression in endometrium	Protein expression in endometrium	Protein expression in endometrium epithelial cells	Protein expression in endometrium stromal cells	Endometriosis status	Affinity energy (kJ/mol)
1NUQ	NMNAT3	Nicotinamide mononucleotide adenylyltransferase 3	7.9	high	high	low	Transcribed	-10
1GZU	NMNAT1	Nicotinamide mononucleotide adenylyltransferase 1	10.1	medium	medium	low	Transcribed	-8.7
1UKI	MAPK8	Mitogen-activated protein kinase 8	17.1	medium	medium	medium	Transcribed	-7.7
1GZ4	ME2	NAD-dependent malic enzyme, mitochondrial	10.5	high	high	low	Transcribed	-7.5
1XO2	CDK6	Cell division protein kinase 6	6	low	low	low	Transcribed	-7.3
2J5F	EGFR	Epidermal growth factor receptor	8.2	low	low	low	Transcribed	-7.3
2FB8	BRAF	B-Raf proto-oncogene serine/threonine-protein kinase	15.3	high	high	medium	Transcribed	-7.2
1YJ6	GSTM1	Glutathione S-transferase Mu 1	9.8	high	high	high	Transcribed	-6.7
1PME	MAPK1	Mitogen-activated protein kinase 1	17.4	medium	medium	medium	Transcribed	-6.5
2B7D	F3	Coagulation factor VII	30.5	low	low	low	Transcribed	-5.9
1A5H	PLAT	Tissue-type plasminogen activator	35.5	low	medium	Low	Transcribed	-5.7
1A4R	CDC42	Cell division control protein 42 homolog	23.9	high	high	medium	Transcribed	-5.2
2VNT	PLAU	Urokinase-type plasminogen activator	10.7	medium	medium	medium	Transcribed	-4.2
2V61	MAOB	Amine oxidase [flavin-containing] B	38	high	high	medium	Transcribed	-0.9
1R1I	MME	Neprilysin	2.8	low	NO	low	Dormant	NA
1LE6	PLA2G10	Group 10 secretory phospholipase A2	0	medium	medium	low	Dormant	NA
1VJ5	EPHX2	Epoxide hydrolase 2	10.3	high	high	NO	NA	NA
1T2F	LDHB	L-lactate dehydrogenase B chain	38.7	high	high	NO	NA	NA
1I72	AMD1	S-adenosylmethionine decarboxylase proenzyme	18.9	low	low	NO	NA	NA
1QH5	HAGH	Hydroxyacylglutathione hydrolase, mitochondrial	6.8	low	low	NO	NA	NA
1Q91	NT5M	5(3)-deoxyribonucleotidase, mitochondrial	2.8	low	low	NO	NA	NA
1GBN	OAT	Ornithine aminotransferase, mitochondrial	21.6	low	low	NO	NA	NA
1V3Q	PNP	Purine nucleoside phosphorylase	5.9	low	low	NO	NA	NA
1GSE	GSTA1	Glutathione S-transferase A1	0.6	NO	Low	NO	NA	NA
1TDI	GSTA3	Glutathione S-transferase A3	0.3	NO	Low	NO	NA	NA
1NHX	PCK1	Phosphoenolpyruvate carboxykinase, cytosolic [GTP]	0.1	low	medium	NO	NA	NA
1PQ3	ARG2	Arginase-2, mitochondrial	3.7	medium	medium	NO	NA	NA
1WMA	CBR1	Carbonyl reductase [NADPH] 1	9.3	medium	medium	NO	NA	NA
1QIP	GLO1	Lactoylglutathione lyase	25.3	medium	medium	NO	NA	NA
1XAN	GSR	Glutathione reductase, mitochondrial	13.2	medium	medium	NO	NA	NA
1MD3	GSTP1	Glutathione S-transferase P	43.5	medium	medium	NO	NA	NA
1LJR	GSTT2B	Glutathione S-transferase theta-2	1.7	medium	medium	NO	NA	NA
3CHO	LTA4H	Leukotriene A-4 hydrolase	27.6	medium	medium	NO	NA	NA
1E8Z	PIK3CG	Phosphatidylinositol-4,5-bisphosphate 3-kinase catalytic subunit gamma isoform	1.7	medium	medium	NO	NA	NA
ALDH2	ALDH2	Aldehyde dehydrogenase, mitochondrial	9	NO	NO	NO	NA	NA
1ELV	C1S	Complement C1s subcomponent	32.2	NO	NO	NO	NA	NA
1LF7	C8G	Complement component C8 gamma chain	0.2	NO	NO	NO	NA	NA
1RS0	CFB	Complement factor B	0.5	NO	NO	NO	NA	NA
1DIC	CFD	Complement factor D	8.9	NO	NO	NO	NA	NA
1T31	CMA1	Chymase	2.9	NO	NO	NO	NA	NA
1T32	CTSG	Cathepsin G	6.2	NO	NO	NO	NA	NA
1PQ2	CYP2C8	Cytochrome P450 2C8	0.1	NO	NO	NO	NA	NA
1OG5	CYP2C9	Cytochrome P450 2C9	0	NO	NO	NO	NA	NA
2P16	F10	Coagulation factor X	12.6	NO	NO	NO	NA	NA
1ZPB	F11	Coagulation factor XI	0.2	NO	NO	NO	NA	NA
1NO9	F2	Prothrombin	0	NO	NO	NO	NA	NA
1ISG	BST1	ADP-ribosyl cyclase 2	6.4	No	No	NO	NA	NA
2VD1	HPGDS	Glutathione-requiring prostaglandin D synthase	4.7	NO	NO	NO	NA	NA
3BZU	HSD11B1	Corticosteroid 11-beta-dehydrogenase isozyme 1	0.8	NO	NO	NO	NA	NA
1PMV	MAPK10	Mitogen-activated protein kinase 10	11	NO	NO	NO	NA	NA
4NOS	NOS2	Nitric oxide synthase, inducible	0.4	NO	NO	NO	NA	NA
1M9R	NOS3	Nitric oxide synthase, endothelial	8.3	NO	NO	NO	NA	NA
PKLR	PKLR	Pyruvate kinase isozymes R/L	0.1	NO	NO	NO	NA	NA
1DCY	PLA2G2A	Phospholipase A2, membrane associated	0.7	NO	NO	NO	NA	NA
2G22	REN	Renin	4.4	NO	NO	NO	NA	NA
1IZ2	SERPINA1	Alpha-1-antitrypsin	0.8	NO	NO	NO	NA	NA
1O4F	SRC	Proto-oncogene tyrosine-protein kinase Src	20.3	NO	NO	NO	NA	NA
1YVL	STAT1	Signal transducer and activator of transcription 1-alpha/beta	17.2	NO	NO	NO	NA	NA
1TFG	TGFB2	Transforming growth factor beta-2	10.8	NO	NO	NO	NA	NA
1RW8	TGFBR1	TGF-beta receptor type-1	18.8	NO	NO	NO	NA	NA

Supplementary Table 4 Profile of genes that are involved in the the KEGG pathway of ProEGCG with top 10 highest enrichment values. Data are sorted in descending order of affinity energies. Only proteins with gene expressions in endometrium, protein expressions in endometrial stromal cells and are transcribed gene in endometriosis patients were proceed for molecular docking. After that, proteins with the most negative affinity energy were selected as final potential protein targets for further chemical binding and functional analysis. [1.] PDB is a 4 characters identification code for each molecular model.
